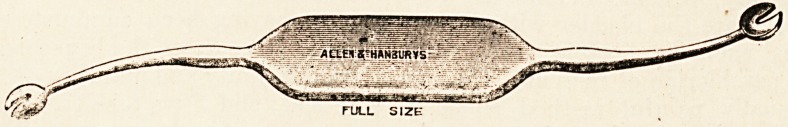# Comedo Extractor

**Published:** 1911-03-11

**Authors:** T. P. Beddoes


### COMEDO EXTRACTOR.

By T. P. BEDDOES, F.R.C.S.

Messrs. Allen and Hanburys have constructed for me a comedo extractor by means of which graduated pressure may be applied whilst the blackhead remains under observation.

The instrument consists of a broad handle with a curved shank at both ends, terminating in a concavo-convex head or dish applicator with a V-shaped nick or slot, the two slots differing in size. The curved shank is so constructed that it adapts itself to all parts of the face. The shape of the slot enables a graduated pressure to be applied exactly around the blocked duct, whilst the slightly concave lower surface of the head allows an even pressure on the skin.

FULL SIZE

The edge of the slot is bevelled and rounded, thus preventing any injury to the skin. The slightly concave upper surface allows each comedo to be removed after extraction quite easily, so that the slot may not become blocked. The method of use is to place the edge of the slot 3 m.m. (one-tenth of an inch) from the comedo and gradually to advance it with increased pressure.

## Figures and Tables

**Figure f1:**